# Multiple-Locus Variable Number Tandem Repeat Analysis for *Streptococcus pneumoniae*: Comparison with PFGE and MLST

**DOI:** 10.1371/journal.pone.0019668

**Published:** 2011-05-26

**Authors:** Karin E. M. Elberse, Sónia Nunes, Raquel Sá-Leão, Han G. J. van der Heide, Leo M. Schouls

**Affiliations:** 1 Laboratory for Infectious Diseases and Perinatal Screening, National Institute for Public Health and the Environment (RIVM), Bilthoven, The Netherlands; 2 Instituto de Tecnologia Química e Biológica, Universidade Nova de Lisboa, Oeiras, Portugal; Swiss Tropical and Public Health Institute, Switzerland

## Abstract

In the era of pneumococcal conjugate vaccines, surveillance of pneumococcal disease and carriage remains of utmost importance as important changes may occur in the population. To monitor these alterations reliable genotyping methods are required for large-scale applications. We introduced a high throughput multiple-locus variable number tandem repeat analysis (MLVA) and compared this method with pulsed-field gel electrophoresis (PFGE) and multilocus sequence typing (MLST). The MLVA described here is based on 8 BOX loci that are amplified in two multiplex PCRs. The labeled PCR products are sized on an automated DNA sequencer to accurately determine the number of tandem repeats. The composite of the number of repeats of the BOX loci makes up a numerical profile that is used for identification and clustering. In this study, MLVA was performed on 263 carriage isolates that were previously characterized by MLST and PFGE. MLVA, MLST and PFGE (cut-off of 80%) yielded 164, 120, and 87 types, respectively. The three typing methods had Simpson's diversity indices of 98.5% or higher. Congruence between MLST and MLVA was high. The Wallace of MLVA to MLST was 0.874, meaning that if two strains had the same MLVA type they had an 88% chance of having the same MLST type. Furthermore, the Wallace of MLVA to clonal complex of MLST was even higher: 99.5%. For some isolates belonging to a single MLST clonal complex although displaying different serotypes, MLVA was more discriminatory, generating groups according to serotype or serogroup. Overall, MLVA is a promising genotyping method that is easy to perform and a relatively cheap alternative to PFGE and MLST. In the companion paper published simultaneously in this issue we applied the MLVA to assess the pneumococcal population structure of isolates causing invasive disease in the Netherlands before the introduction of the 7-valent conjugate vaccine.

## Introduction

*Streptococcus pneumoniae* is an important human pathogen causing diseases like otitis, pneumonia, sepsis, and meningitis. The major virulence factor of the pneumococcus is the capsule [1], [2]. Currently, over 90 different capsules have been identified [3], [4], [5], [6]. Such high antigenic diversity has long been recognized and serotyping using Quellung reaction has been used for pneumococcal typing for several decades. In recent years, as the DNA sequences of the capsular biosynthetic loci became available [5], [7], alternative strategies for capsular typing based on genotyping methods have been developed [8], [9], [10] (Elberse et al. companion paper, PLoS One, this issue).

For the past two decades, a number of genotyping methods aimed to assess the genetic diversity of pneumococcal isolates have been used. Currently, pulsed-field gel electrophoresis (PFGE) [11], [12] and multilocus sequence typing (MLST) [13] are the gold standards for genotyping of pneumococci.

In PFGE, total DNA is digested with a rare cutter endonuclease (such as *Sma*I or *Apa*I) that yields a limited number of fragments of high molecular weight. The fragments are separated by a variant of gel electrophoresis in which the orientations of the electric field change periodically, enabling megabase size DNA fragments to be effectively separated by size [14]. The DNA banding patterns are then compared between isolates and clonal relationships are inferred [12]. Although the interpretation criteria may vary depending on the size of the collection and on the goal of the research being conducted, there are general criteria, both visual and computer-assisted that seem to work well [15], [16]. Advantages of PFGE are that it has good typeability (the percentage of isolates that can be assigned a type), reproducibility, and resolving power [16], [17]. In addition, the costs for materials and equipment are relatively low and handling of the equipment is easy. However, it is laborious and time consuming and may yield ambiguous results if not performed by a well trained technician. PFGE is quite useful for local epidemiology and it has also been used for global epidemiology once standardized [17], [18]. Portability between laboratories is not straightforward, but seems to be attainable provided protocol harmonization is achieved [16].

Multilocus sequence typing (MLST) is a DNA sequence-based method that relies on PCR amplification and sequencing of internal fragments of 7 housekeeping genes [13], [19]. For allele assignment, each sequence is compared to all known alleles which are available at an online database (www.mlst.net). Different sequences (even single nucleotide difference) are assigned different allelic numbers. The 7 assigned allele numbers form an allelic profile or sequence type (ST). MLST is expensive if in-house sequencing facilities are not available and, therefore, many laboratories cannot afford to use it routinely. However, it has the advantages of being reproducible, unambiguous, portable allowing intra-laboratory comparisons and suited to create international databases. For *S. pneumoniae*, MLST has a good resolving power being useful for local and global epidemiology. Furthermore, in contrast to PFGE, MLST does not always require a culture and can sometimes be directly performed on samples containing bacterial DNA such as cerebrospinal fluid [20].

In 1992 conserved repeated sequences, named BOX elements, were identified in the genome of the pneumococcus [21]. The sequenced genomes of R6 and TIGR4 contain 115 and 127 BOX elements, respectively. BOX elements consist of 3 different subunits, BoxA, BoxB and BoxC. BoxB is the tandem repetitive unit of 45 base pairs flanked by BoxA and BoxC, although elements missing BoxA or missing BoxC have been described [21], [22]. The function and origin of BOX elements are unknown; however, it is thought that they may be involved in regulating the expression of virulence-associated genes when they are located in the promoter regions of genes [21], [22]. In the years after the discovery of these elements a number of genotyping methods were introduced based on the variability of these elements. A BOX-PCR described by van Belkum and colleagues showed the usefulness of these repeats for genotyping [23]. The primer of this PCR is based on the BoxA sequence and yields PCR products of regions present between BOX loci that are close to each other and in opposite direction. However, the method has the disadvantage of creating banding profiles, which are difficult to interpret and often lack reproducibility. A multiple-locus variable number tandem repeat analysis (MLVA) scheme based on BOX typing was introduced by Koeck et al. in 2005 [24]. It analyzes 16 BOX loci that are PCR-amplified in single PCR reactions and products are analyzed by agarose gel electrophoresis. A website (www.mlva.eu) providing a database in which profiles can be compared has been created. At the time of writing this manuscript the MLVA profiles of 1147 isolates had been deposited in the database. The many loci that are analyzed and the choice of agarose gel electrophoresis for sizing makes this method somewhat laborious. This MLVA scheme was successfully applied and compared with MLST and was shown to yield high congruence with MLST, but was more discriminatory than MLST [25], [26].

In this paper we describe a newly developed MLVA typing scheme for pneumococci based on 8 BOX elements. The 8 BOX loci are amplified in 2 multiplex PCRs and the fluorescently labeled PCR products are sized on an automated sequencer yielding high throughput and unambiguous typing results. The generated MLVA results can be compared internationally using the newly available MLVA website www.MLVA.net. On the website the MLVA profiles can be imported to assess the MLVA type (MT). In the current study we assess the validity of this method by comparing MLVA with two well established genotyping methods: PFGE and MLST. In the companion paper published simultaneously in PLoS One, we applied this MLVA scheme to assess the population structure of pneumococci causing invasive disease in the Netherlands before the introduction of the 7-valent pneumococcal conjugate vaccine.

## Results

### Multiple-locus variable number tandem repeat analysis (MLVA)

A combination of highly diverse and less diverse BOX loci were chosen aiming to have a good balance between loci that would evolve faster than others. The 13 randomly chosen BOX loci were tested on a panel of 84 isolates and 8 BOX loci were chosen for the MLVA scheme. The overlap in BOX loci of our MLVA scheme with the MLVA scheme previously described by Koeck et al. [24] is provided in Table 1. In particular, BOX loci 1, 4, 12 and 13 were rather diverse while BOX locus 11 had less diversity. If BOX loci could not be amplified they were assigned allele number 99. To exclude the possibility that BOX loci could change by laboratory storage and repeated subculture, 4 pneumococcal isolates were subcultured for 29 sequential days. MLVA was performed on samples from days 1, 15, and 29 and no changes in the number of repeats in the 8 BOX loci were observed indicating that the composition of BOX loci remained stable under laboratory conditions (data not shown). In addition, 84 isolates were typed at least twice at different occasions by independent technicians and the same MLVA types (MT) were obtained indicating that the MLVA scheme is reproducible.

**Table 1:** Oligonucleotide primer sequences used in *S. pneumoniae* MLVA scheme.

Assay	Primer	Primer sequence	Accession no.	Coordinates	Koeck et al.
Multiplex reaction 1	BOX_01-Ff^1^	CCAGAGACATTGATGAAGAGA	NC_003098	1611150	Spneu40
	BOX_01-r	CGCTTTGATGAACTTGAGTT	NC_003098	1611477	Spneu40
	BOX_02-Nf^1^	TTGCTTGGTACAGAAAACAAA	NC_003098	571757	Spneu32
	BOX_02-r	CCCCATAAAACCCTCCTTATA	NC_003098	572108	Spneu32
	BOX_03-Vf^1^	TCCAACACGACCTTTATCC	NC_003098	1579329	Spneu15
	BOX_03-r	TTCAGTAAACCCAGCTCGTA	NC_003098	1579997	Spneu15
	BOX_04-f	TGGGTAAAAGTAGACAGGACT	NC_003098	698447	Spneu33
	BOX_04-f2^2^	AGGGGATTTACCCACTACAAA	CP000936	823993	
	BOX_04-Pr^1^	CACTTCTACACTAGTTTGTAAAGCA	NC_003098	698665	Spneu33
Multiplex reaction 2	BOX_06-Nf^1^	GAAAAAGGTCAGGAGTAGGTT	NC_003098	1911454	Spneu38
	BOX_06-r	TCACTTGAGACAATCAGCC	NC_003098	1911736	Spneu38
	BOX_06-Nr2^1^^,^ 2	GAAATCTTTGAAAAACTAGGATTT	NC_003098	1911683	Spneu38
	BOX_06-f2^2^	TTATGATTTTTGTCATTTTGCA	NC_003098	1911429	Spneu38
	BOX_11-Vf^1^	TCCAATAATGACAGGTTTTCCTC	NC_003098	411593	
	BOX_11-r	TTCCAATCTACGCCTTTGAAG	NC_003098	412169	
	BOX_12-Pf^1^	TTGCCCTTTTCATCTTCGA	NC_003098	1350183	Spneu37
	BOX_12-r	CAGCAACCATTGAAACGC	NC_003098	1350824	Spneu37
	BOX_13-Ff^1^	TCGCCTTTGCTAATCAAACA	NC_003098	101033	Spneu25
	BOX_13-r	CTGATTATATCGCTCACAAACCC	NC_003098	101490	Spneu25

1 Primer is fluorescently labeled. F: FAM; N: NED; V: VIC; P: PET.

2 Additional primer because of polymorphism in primer sites.

### Discriminatory power of MLVA

The discriminatory power of MLVA was calculated by using the Simpson's Index of Diversity (SID) applied to the test population. The 263 isolates were resolved in 164 types that contained between 1 and 14 isolates. The SID for MLVA was 0.993, a very high value comparable to the ones obtained for PFGE (0.985) and MLST (0.987) (Table 2). In Table S1 the MLVA, MLST and PFGE data is provided for all isolates that are included in this study (Table S1). The companion paper (Plos One, this issue) highlights its wide application to over 1000 isolates, including 444 MLVA types. Overall, the current MLVA database consists of 2973 isolates, 960 MLVA types and 43 MLVA complexes. The SID for MLVA based on the entire database was 0.986 (95% CI, 0.985–0.988) (www.MLVA.net, last accessed on October 20, 2010).

**Table 2:** Simpson's Index of Diversity of the different typing methods.

Typing method	SID^1^ [95% CI]	No. of types
Serotyping	0.937 [0.926–0.948]	41
PFGE	0.985 [0.983–0.988]	87
MLST	0.987 [0.981–0.992]	120
MLST_CC^2^	0.963 [0.955–0.971]	56
MLVA	0.993 [0.991–0.996]	164
MLVA_MC^3^	0.935 [0.918–0.953]	25

1 SID, Simpson's Index of Diversity; 95% CI, 95% confidence interval.

2 CC, clonal complex.

3 MC, MLVA complex.

### Comparison of MLVA with serotyping, PFGE, and MLST

To assess the congruence between typing methods the Wallace index was calculated (Table 3). This coefficient indicates the probability that a pair of isolates which is assigned to the same type by one typing method is also typed as identical by the other method. A very good directional correlation between MLVA and MLST results was found: the probability of two isolates having the same MLVA type (MT) also sharing the same MLST type (ST) was 87.4%. This value was even higher for predicting the MLST clonal complex: 99.5%. Corresponding Wallace indexes between MLVA and other typing methods were: 82.2% for serotyping, and 46.5% for PFGE. By contrast, the chance that two isolates sharing the same ST also shared the same MT was 43.3% and sharing the same MLVA complex was 99.2%. Lower values were obtained for PFGE and serotyping. These differences can be explained by the higher discriminatory power of MLVA compared to the other typing methods.

**Table 3:** Congruence between typing methods expressed by Wallace coefficients.

Wallace coefficient [95% CI]^1^						
Typing method	Serotype	PFGE	MLST	MLST_CC^2^	MLVA	MLVA_MC^3^
Serotype		0.091 [0.064–0.118]	0.187 [0.132–0.242]	0.343 [0.279–0.407]	0.087 [0.053–0.122]	0.631 [0.553–0.710]
PFGE	0.386 [0.329–0.442]		0.325 [0.273–0.376]	0.550 [0.493–0.607]	0.209 [0.157–0.261]	0.718 [0.649–0.786]
MLST	0.875 [0.848–0.902]	0.358 [0.276–0.439]		1.000 [1.000–1.000]	0.433 [0.316–0.550]	0.992 [0.983–1.001]
MLST_CC^2^	0.575 [0.526–0.623]	0.232 [0.180–0.284]	0.419 [0.356–0.481]		0.202 [0.140–0.263]	0.881 [0.849–0.912]
MLVA	0.822 [0.779–0.865]	0.465 [0.396–0.535]	0.874 [0.825–0.923]	0.995 [0.991–1.000]		1.000 [1.000–1.000]
MLVA_MC^3^	0.769 [0.717–0.822]	0.295 [0.224–0.366]	0.582 [0.486–0.679]	0.998 [0.997–1.000]	0.295 [0.199–0.391]	

1 95% CI, 95% confidence interval.

2 CC, clonal complex.

3 MC, MLVA complex.

### Relatedness of isolates with similar types when assessed by MLVA or MLST

**Figure 1:**
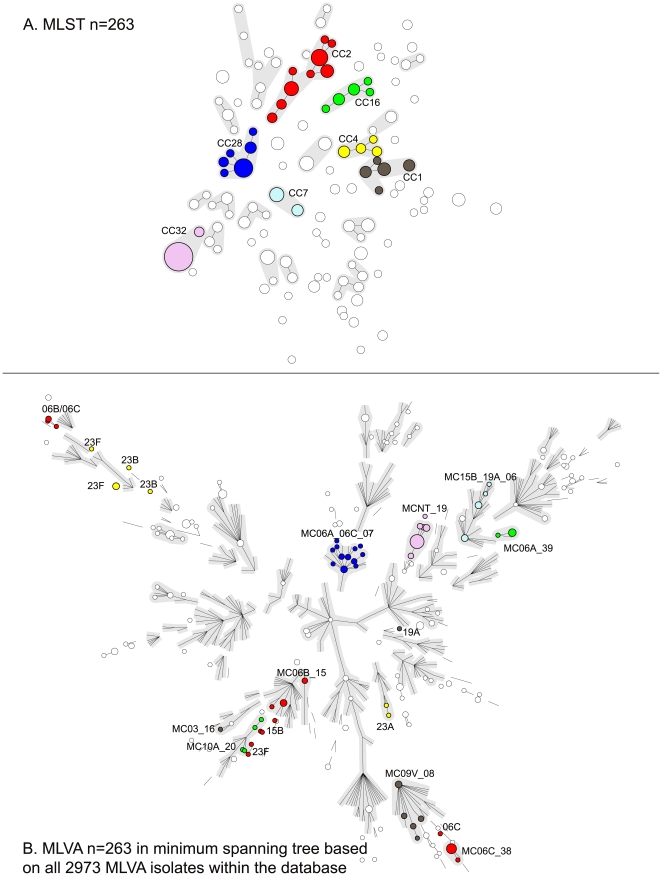
High congruence between MLVA and MLST. Minimum spanning tree of the results obtained by MLST (A) and MLVA (B) for the 263 isolates of the test collection. Each circle indicates a genotype. The size of the circle is proportional to the number of isolates with the same type. Lines linking two types denote a single locus difference between those types. In Figure 1A the MLST complexes are indicated by halos. Seven MLST complexes are discussed in the manuscript and these are colored and named according to the nomenclature of *S. pneumoniae* MLST database (www.mlst.net, last accessed on October 20, 2010). The white nodes represent the isolates within this test collection that are not discussed. The same colors were applied to the minimum spanning tree based on MLVA in Figure 1B. This minimum spanning tree is based on the entire MLVA database (last accessed on October 20, 2010), and only the branches are made visible. The MLVA types of the 263 isolates used in this study were depicted as circles. The halos indicate the MLVA complexes.

Although there was considerable concordance between MLST and MLVA, some of the isolates that were grouped by MLST could be further distinguished by MLVA. In Figure 1A a minimum spanning tree is depicted based on the MLST results for the 263 isolates of the test collection. Fifty-six clonal complexes (CC), and 13 singletons were detected and the 7 largest CCs were chosen for further comparisons and are highlighted in color. To visualize the relationship between MLST and MLVA, the same color legend was applied to the minimum spanning tree based on MLVA (Fig. 1B). To create this minimum spanning tree, the entries in the entire MLVA database (n = 2973) were used. Subsequently, a subnetwork was generated displaying the branches and complexes of the complete tree but only the nodes representing the isolates used in this study.

MLST CC28 included isolates with serotype 6A and 6C which were also closely related when characterized by MLVA. Similar results were obtained for MLST CC1, CC7 and CC32. CC16 included isolates of serotypes 6A, 6C, and 10A; isolates of serotype 10A were clearly separated by MLVA from the serotype 6A and 6C isolates. CC2 consisted of serotype 6A, 6B, 6C, 15B and 23F isolates. The serotype 6A, 6B and 6C isolates were separated in 2 distinct groups when they were characterized by MLVA. The 15B and 23F isolates were clearly distinct and yielded completely different MTs. This was an example were MLVA distinguished isolates with different serotypes that were grouped within a single CC. The same could be observed for MLST CC4 that comprised serotypes 23A, 23B and 23F that were dispersed into 2 groups using the MLVA.

In this test collection there were 9 instances where isolates were characterized by MLST as two different types, whereas by MLVA these isolates yielded the same type. These isolates were all single locus variants, except for a single isolate.

## Discussion

In recent years, typing schemes based on MLVA have been designed and implemented for a number of micro-organisms of public health importance, including *Bacillus anthracis*, *Staphylococcus aureus*, *Enteroccus faecium*, *Haemoplhilus influenza*, *Bordetella pertussis* and many others [27], [28], [29], [30], [31], [32], [33], [34], [35]. MLVA-based typing strategies have several appealing characteristics that match the convenience criteria desirable for typing methods [36]. For example, once implemented MLVA requires minimal technical expertise and typing results can be obtained in a few hours providing a sequencing facility is readily available. The equipment needed is the same as for MLST, i.e., a thermocycler and a DNA sequencer, equipment that many laboratories performing typing have already access to. It is relatively low cost (cheaper than MLST) and the data are amenable to automatic analysis by computer software (as in MLST). Furthermore, the results are unambiguous numerical profiles, portable, and thus suitable for intra-laboratory comparisons. In addition, PFGE can only be performed on bacterial cultures while MLST can be performed on clinical samples containing bacterial DNA such as cerebrospinal fluid [20]. The MLVA has the same potential as MLST, although this has not been described in detail yet.

In this study we assessed the validity of a MLVA strategy developed for *Streptococcus pneumoniae* using as a test collection over 263 pneumococcal isolates previously characterized by the three gold-standard pneumococcal typing methods: serotyping, PFGE, and MLST. The scheme explored polymorphisms scattered throughout the genome of R6 focusing in eight BOX loci for size estimation. The amplification of the 8 loci was combined in two multiplex PCR reactions. Fragment sizing was performed automatically on a DNA sequencer, resulting in a fast, easy and accurate interpretation of results.

The MLVA for the pneumococcus was first described by Koeck et al. [24]. In that MLVA scheme, 16 BOX loci were used and analyzed by agarose gel electrophoresis. Remarkably, 7 of the 8 BOX loci used in our MLVA scheme overlap with loci included in the previously published MLVA. We started the selection of the loci for our MLVA already in 2005, before the publication of the MLVA by Koeck et al. and thus the selection was performed independently. Therefore, the overlap is coincidental, but suggests the proper choice of a combination of more and less diverse loci in both MLVA schemes was made. The main advantage of our MLVA is the high throughput that is facilitated by the use of two multiplex assays instead of 16 separate reactions as done by Koeck et al. and the use of capillary electrophoresis for sizing. Furthermore, the electronic data management that we propose makes it easier to analyze the data than images of bands obtained by agarose gel electrophoresis. We restricted our MLVA to 8 VNTR loci to limit the amount of work required to type an isolate. Our results show that this scheme yields a high resolution. The resolution will probably be even higher if more loci were used like in Koeck's MLVA scheme. However, this will depend on the questions that need to be answered. For our studies MLVA should be utilized to determine the genetic background of isolates and relate this to changes in other properties such as capsular composition. In that case MLVA based on 8 loci seems to suffice. Of note, both MLVA schemes showed a high congruence with MLST [25], [26].

Although the test collection included only carriage isolates, we do not consider it a limitation to the validation study for the following reasons: (i) it is a very diverse collection that includes epidemiologically unrelated, as well as closely-related isolates; (ii) it is well known that there are substantial overlaps among isolates being carried and causing disease at a given time and geographic location although the relative frequency in each group may vary significantly [18]; (iii) this MLVA scheme has in the mean time been successfully applied to a collection of over 1000 invasive disease isolates from all over the Netherlands (Elberse et al. companion manuscript, PLoS One, this issue). In addition, in a manuscript in preparation we will use the MLVA to assess temporal changes and vaccine related changes in the genetic background of the pneumococcus.

We found that the MLVA scheme described here met all the performance criteria needed for a good typing method [36]: it was stable, typed 100% of the isolates, was reproducible and had a high discriminatory power. Of interest, the congruence between MLVA and MLST was very good suggesting it can be routinely used as an alternative technique to MLST. Furthermore, MLVA was able to discriminate between putative capsular variants sharing the same MLST in a number of occasions suggesting an enhanced epidemiological usefulness. To make this MLVA scheme easily available to the typing community, an electronic pneumococcal MLVA database located at www.MLVA.net has been created. This application contains the detailed protocol and materials needed for implementation of the MLVA scheme, contact information to obtain control samples, an application for MLVA assignment, and a database for international deposition of MLVA types.

In summary, the MLVA scheme for pneumococci proposed in this study has the convenience and performance criteria needed for a good typing method. It has the advantages of being portable as MLST but is faster and has lower costs. The high congruence between MLVA and MLST suggests it can be applied as a general methodology and, if needed, MLST may be performed on selected isolates from clusters sharing related MT. In the era of pneumococcal conjugate vaccines, close surveillance of pneumococcal populations is of crucial importance; this MLVA scheme represents a high-throughput typing technique which combines easiness, speed, low cost, and portability and therefore can be a rather useful tool to achieve such goal.

## Materials and Methods

### Isolate collection

In this study 263 pneumococcal isolates were used for the comparison of MLVA with PFGE and MLST. The isolates were obtained between 1996 and 2007 from the nasopharynx of children attending several day-care centers in the area of Lisbon, Portugal. The isolates were stored in glycerol broth at −80°C and were characterized by antibiogram, serotyping, PFGE, and MLST under the scope of previous studies [18], [37], [38]. Approval for the study was obtained from the Ministry of Education and from the directors of each day-care center. Written informed consent was obtained from parents or guardians of each child before sampling. All information was stored in an in-house developed online database. Overall, the collection comprised 41 serotypes, 87 PFGE types and 120 STs.

### Serotyping, PFGE and MLST

These techniques were performed as previously described. Briefly, serotyping was performed by the Quellung reaction [39] and/or multiplex PCR [40], [41]. For PFGE, total DNA was extracted, restricted with *Sma*I, and DNA fragments were resolved by PFGE [18]. MLST was performed using primers and conditions described previously [13]. For allele assignment the *S. pneumoniae* MLST database available at www.mlst.net was interrogated. Sequence traces of novel alleles were submitted to the curator for new allele and ST assignment.

### Multiple-Locus Variable number tandem repeat Analysis (MLVA)

**Figure 2:**
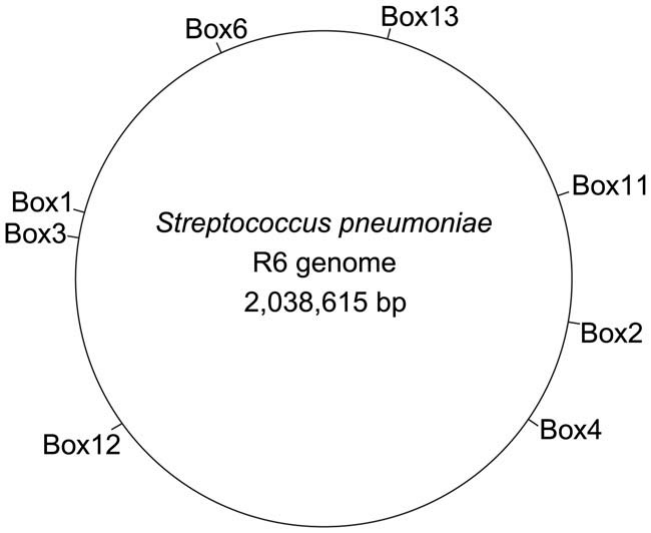
Schematic representation of the pneumococcus R6 genome indicating the location of the 8 BOX loci that are used in the MLVA scheme.

In the genome sequence of isolate R6 (GenBank number NC_003098), one of the publicly available genomes, 127 BOX tandem repeats were found. Thirteen randomly chosen BOX loci scattered throughout the R6 genome to be used in the MLVA scheme were tested on a panel of 84 isolates. Eight BOX loci were chosen and primers were designed targeting the flanking regions of the BoxA and BoxC elements (Figure 2, Table 1). One primer of each primer set was fluorescently labeled with FAM, NED, VIC or PET. For BOX loci 4 and 6 respectively 3 and 4 primers were used to enhance amplification within all isolates. The 8 loci were amplified in 2 separate multiplex PCRs, creating mixtures each with 4 different fluorescent labels enabling analysis of 4 BOX loci in a single fragment analysis reaction (Table 1). Amplification of the loci was done in Applied Biosystems 9700 PCR machines. The 25 µl PCR reaction mixtures consisted of Qiagen multiplex PCR mix, 10 µM of each primer and 2 µl of 1∶10 lysates diluted in sterile water. Lysates were prepared by suspending a loop full of pneumococci in 500 µl TE (10 mM Tris.HCl and 1 mM EDTA, pH 8) followed by heating for 10 minutes by 95°C. Amplification was performed using the following PCR program: 15 min 95°C, 25 cycles of 30 sec 95°C, 1 min 54°C and 1 min 72°C followed by a 30 min incubating at 68°C to ensure complete addition of the extra 3′adenosin by the terminal transferase activity of the Taq polymerase. Of the PCR product mixtures, 2 µl aliquots, diluted 1∶200 in water, were mixed with 10 µl of 1∶200 diluted Genescan 1200 LIZ-marker (Applied Biosystems, Foster City, U.S.A.). The product was heated for 5 minutes at 95°C for denaturation and sized on the AB 3730 DNA sequencer using the Fragment Analysis module.

Determination of the number of repeats in each locus was done using the GeneMarker software (Softgenetics, State College, Pennsylvania, USA). For this purpose, fragment analysis files with the .fsa extension were imported into the software and the number of repeats in each locus was derived from the data. All alleles that yielded PCR product sizes that had not been found before, were analyzed by sequencing using unlabeled versions of the MLVA primers. This included PCR products with aberrant sizes representing loci in which deletions or insertions had occurred in the region flanking the BOX repeats. Thereafter, the loci were assigned the number of BoxB repeats present. For each isolate an allelic profile was generated consisting in a string of 8 integers reflecting the number of repeats in the 8 BOX loci. The allelic profile was assigned an arbitrary sequential MLVA type (MT). The correspondence between MT and allelic profile was deposited at the MLVA database located at www.mlva.net to guarantee that the same nomenclature was maintained for all isolates characterized by this technique regardless of its origin and study.

### Data Analysis

Analysis of PFGE patterns, MLST and MLVA numerical profiles, was performed using Bionumerics version 6.1 (Applied Maths, Sint-Martens-Latem, Belgium).

PFGE patterns were clustered by UPGMA. A dendrogram was generated from a similarity matrix calculated using the Dice similarity coefficient with an optimization of 1.0% and a tolerance of 1.5%. PFGE clusters were defined as isolates with a similarity of 80% or higher on the dendrogram [38], [42].

Clustering of MLST and MLVA types were displayed in minimum spanning trees. In this graphic representation, the circles in the tree represent the various types. The size of the circle is proportional to the number of isolates with the same type. Lines linking two types in the tree denote single locus difference between those types. For assignment of MLST or MLVA clonal complex, the entire MLST database (available at www.mlst.net, last accessed on October 20, 2010) or the entire in-house MLVA database (available at www.mlva.net, last accessed on October 20, 2010) were interrogated. MLVA complex assignment was based on a maximum distance of one locus between related types. The minimum number of MLVA types in a MLVA complex was set to 3 with a minimum of 9 entries per MLVA complex, resulting in on average 3 isolates within an MLVA type. These settings resulted in MLVA complexes that were strongly correlated to serotype.

### Statistical analysis

The discriminatory ability of the three typing systems - PFGE, MLST and MLVA - was measured using the Simpson's index of diversity (SID) and 95% confidence intervals were calculated as proposed before [43], [44], [45]. The congruence between the typing methods was calculated using the Wallace coefficient [46]. All calculations were done using the freely available online tool Comparing Partitions located at www.comparingpartitions.info [46].

## Supporting Information

Table S1 **MLVA, MLST and PFGE results for all isolates included in this study (n = 263).**
(PDF)
