# Supplementary material for: Multiple-Locus Variable Number Tandem Repeat Analysis for Streptococcus pneumoniae: Comparison with PFGE and MLST
Source: PLoS One. 2011 May 26;6(5):e19668. doi: 10.1371/journal.pone.0019668 (PMC3102655; doi:10.1371/journal.pone.0019668)
Supplement: Table S1 — MLVA, MLST and PFGE results for all isolates included in this study (n = 263). (PDF) [file pone.0019668.s001.pdf]

					MLVA profiles, BOX locus								MLST profiles						
Key	Serotype	MT	ST	PFGE	1	2	3	4	6	11	12	13	aroe	gdh	gki	recP	spi	xpt	ddl
PT5574	01	261	306	42	2	3	2	4	3	1	7	4	12	8	13	5	16	4	20
PT5037	01	261	306	42	2	3	2	4	3	1	7	4	12	8	13	5	16	4	20
PT5582	01	261	306	42	2	3	2	4	3	1	7	4	12	8	13	5	16	4	20
PT5279	01	256	228	50	2	3	2	4	2	1	7	8	12	8	1	5	17	4	20
PT560	01	210	304	13	2	2	9	4	1	1	4	4	13	8	13	5	17	4	8
PT500	03	339	162	59	3	1	2	2	1	2	99	5	13	11	10	1	6	8	14
WL812	03	423	1230	16	3	3	2	3	2	1	1	3	7	15	2	10	6	1	144
PT4914	03	427	180	16	3	3	2	4	2	1	1	3	7	15	2	10	6	1	22
PT4076	03	427	180	16	3	3	2	4	2	1	1	3	7	15	2	10	6	1	22
WL696	03	251	180	38	2	3	2	3	2	1	1	3	7	15	2	10	6	1	22
WL1135	03	251	1230	63	2	3	2	3	2	1	1	3	7	15	2	10	6	1	144
PT1263	03	340	232	59	3	1	2	2	2	2	99	5	13	9	15	14	10	16	1
PT2537	04	656	1221	32	5	2	16	4	2	2	2	1	7	5	1	5	15	12	14
PT10	06A	382	1876	14	3	2	5	4	2	2	7	2	7	25	4	12	15	20	28
PT457	06A	382	1876	15	3	2	5	4	2	2	7	2	7	25	4	12	15	20	28
PT1696	06A	379	1152	24	3	2	4	4	99	2	7	2	2	13	2	4	9	157	1
PT5659	06A	642	1098	51	5	2	7	4	3	2	7	2	7	25	4	1	15	3	28
PT1583	06A	578	681	13	4	3	12	5	2	1	1	5	2	5	9	1	6	19	14
PT2527	06A	125	1880	26	1	3	7	3	3	1	4	3	5	7	4	1	10	1	27
WL70	06A	66	65	26	1	2	7	3	3	1	4	3	2	7	4	10	10	1	27
PT5401	06A	125	460	26	1	3	7	3	3	1	4	3	5	7	4	10	10	1	27
PT3831	06A	430	1150	5	3	3	4	2	3	2	3	6	7	25	8	6	25	6	8
PT1304	06A	430	1150	5	3	3	4	2	3	2	3	6	7	25	8	6	25	6	8
PT2628	06A	363	176	7	3	2	2	3	1	2	6	6	7	13	8	6	10	6	14
PT1699	06A	412	1878	103	3	3	1	1	2	1	5	1	7	8	1	1	15	178	14
PT2399	06A	412	1878	55	3	3	1	1	2	1	5	1	7	8	1	1	15	178	14
WL613	06A	751	327	81	6	3	12	99	2	1	1	1	1	5	7	12	10	1	14
PT320	06A	689	327	81	5	3	12	99	2	1	1	1	1	5	7	12	10	1	14
PT3651	06A	689	327	81	5	3	12	99	2	1	1	1	1	5	7	12	10	1	14
WL1264	06A	752	1714	83	6	3	12	99	2	1	1	6	1	5	7	12	17	148	14
PT1175	06A	774	273	36	7	2	6	3	1	2	5	2	5	6	1	2	6	1	14
PT4937	06A	388	1879	23	3	2	6	4	2	2	7	4	7	25	54	4	117	20	28
WL215	06A	727	2191	20	6	2	10	6	1	2	1	2	2	2	100	1	15	20	8
PT2407	06A	388	1879	23	3	2	6	4	2	2	7	4	7	25	54	4	117	20	28
PT2703	06B	455	469	17	3	3	11	3	1	2	9	2	7	13	27	6	10	6	14

Key	Serotype	MT	ST	PFGE	MLVA profiles, BOX locus								MLST profiles						
					1	2	3	4	6	11	12	13	aroe	gdh	gki	recP	spi	xpt	ddl
PT2743	06B	453	469	17	3	3	10	3	1	2	9	2	7	13	27	6	10	6	14
PT289	06B	521	1078	6	4	2	2	3	1	2	6	6	7	8	8	6	10	6	14
PT4058	06B	521	176	7	4	2	2	3	1	2	6	6	7	13	8	6	10	6	14
PT3572	06B	521	176	7	4	2	2	3	1	2	6	6	7	13	8	6	10	6	14
PT3487	06B	363	176	7	3	2	2	3	1	2	6	6	7	13	8	6	10	6	14
PT211	06B	354	887	103	3	1	8	1	2	1	5	1	20	28	1	1	76	14	14
PT1605	06B	354	887	55	3	1	8	1	2	1	5	1	20	28	1	1	76	14	14
PT3919	06B	352	887	55	3	1	7	1	2	1	5	1	20	28	1	1	76	14	14
PT5054	06B	354	887	55	3	1	8	1	2	1	5	1	20	28	1	1	76	14	14
PT76	06B	776	273	12	7	2	6	3	3	2	1	2	5	6	1	2	6	1	14
DCC2613	06B	776	90	13	7	2	6	3	3	2	1	2	5	6	1	2	6	3	4
PT1430	06B	776	273	36	7	2	6	3	3	2	1	2	5	6	1	2	6	1	14
PT2236	06B	766	90	36	7	2	2	3	3	2	1	2	5	6	1	2	6	3	4
PT3104	06B	814	90	36	8	2	2	3	3	2	1	2	5	6	1	2	6	3	4
PT3536	06B	776	273	36	7	2	6	3	3	2	1	2	5	6	1	2	6	1	14
DCC2623	06B	821	90	36	9	2	6	3	3	2	1	2	5	6	1	2	6	3	4
PT582	06B	776	273	36	7	2	6	3	3	2	1	2	5	6	1	2	6	1	14
DCC2160	06B	401	896	34	3	2	9	3	2	2	1	11	7	62	1	2	36	118	14
PT51b	06B	777	885	8	7	2	6	3	3	2	1	4	1	6	1	2	75	1	14
PTRL11007	06B	401	896	34	3	2	9	3	2	2	1	11	7	62	1	2	36	118	14
PT4036	06C	382	1876	14	3	2	5	4	2	2	7	2	7	25	4	12	15	20	28
PT1769	06C	382	1876	51	3	2	5	4	2	2	7	2	7	25	4	12	15	20	28
PT2100	06C	382	1876	51	3	2	5	4	2	2	7	2	7	25	4	12	15	20	28
PT6257	06C	635	2185	35	5	2	5	1	2	2	4	6	1	10	9	43	5	1	6
PT2030	06C	125	460	45	1	3	7	3	3	1	4	3	5	7	4	10	10	1	27
PT2297	06C	125	460	46	1	3	7	3	3	1	4	3	5	7	4	10	10	1	27
PT5442	06C	454	3673	17	3	3	10	3	1	2	9	3	1	13	27	6	10	6	14
PT6119	06C	454	3673	19	3	3	10	3	1	2	9	3	1	13	27	6	10	6	14
PT4913	06C	738	2689	5	6	3	4	2	3	1	3	6	7	25	8	6	25	28	8
PT4542	06C	430	1150	5	3	3	4	2	3	2	3	6	7	25	8	6	25	6	8
PT4291	06C	430	3672	5	3	3	4	2	3	2	3	6	1	25	8	6	25	6	8
PT3371	06C	430	1150	5	3	3	4	2	3	2	3	6	7	25	8	6	25	6	8
DCC1100	06C	430	1150	5	3	3	4	2	3	2	3	6	7	25	8	6	25	6	8
PT449	06C	430	1150	5	3	3	4	2	3	2	3	6	7	25	8	6	25	6	8
DCC851	06C	431	1150	52	3	3	4	2	99	2	3	6	7	25	8	6	25	6	8

Key	Serotype	MT	ST	PFGE	MLVA profiles, BOX locus								MLST profiles						
					1	2	3	4	6	11	12	13	aroe	gdh	gki	recP	spi	xpt	ddl
PT2642	06C	625	176	6	5	2	2	3	1	2	6	6	7	13	8	6	10	6	14
PT5785	06C	752	1714	42	6	3	12	99	2	1	1	6	1	5	7	12	17	148	14
PT3016	06C	753	395	48	6	3	13	99	2	1	1	6	1	5	7	12	17	1	14
PT2228	06C	735	3218	50	6	3	3	8	2	1	1	6	1	5	7	12	10	1	8
PT2317	06C	690	395	82	5	3	12	99	2	1	1	2	1	5	7	12	17	1	14
DCC1353	06C	693	395	83	5	3	12	99	6	1	2	1	1	5	7	12	17	1	14
DCC1016	06C	691	395	83	5	3	12	99	2	1	1	6	1	5	7	12	17	1	14
PT5019	06C	691	395	84	5	3	12	99	2	1	1	6	1	5	7	12	17	1	14
PT6298	06C	654	1692	84	5	2	12	99	2	1	1	6	1	5	7	12	17	158	14
PT4482	06C	694	395	84	5	3	13	99	2	1	1	6	1	5	7	12	17	1	14
DCC2420	06C	694	395	84	5	3	13	99	2	1	1	6	1	5	7	12	17	1	14
DCC333	06C	696	3711	84	5	3	13	99	2	99	1	6	1	5	7	12	17	1	307
PT4252	06C	695	395	84	5	3	13	99	2	1	1	7	1	5	7	12	17	1	14
PT6262	06C	691	395	85	5	3	12	99	2	1	1	6	1	5	7	12	17	1	14
PT5315	06C	160	3396	59	2	1	8	1	2	1	2	4	32	28	1	1	15	16	14
PT6288	06C	160	3396	60	2	1	8	1	2	1	2	4	32	28	1	1	15	16	14
DCC2182	06C	203	2789	44	2	2	8	2	1	2	2	4	7	60	8	5	6	19	14
DCC2584	06C	728	3671	52	6	2	10	10	2	2	7	1	7	25	4	12	6	1	28
PT3354	07F	468	191	43	3	4	2	1	0	1	1	2	8	9	2	1	6	1	17
WL184	07F	468	191	43	3	4	2	1	0	1	1	2	8	9	2	1	6	1	17
PT5303	07F	468	191	54	3	4	2	1	0	1	1	2	8	9	2	1	6	1	17
PT4216	07F	95	1766	29	1	2	11	3	1	1	2	2	1	5	29	1	46	14	18
PT4217	08	18	53	13	1	2	1	4	1	1	2	1	2	5	1	11	16	3	14
PT2533	09V	817	162	28	8	3	4	1	2	1	3	2	7	11	10	1	6	8	14
PT494	09V	277	162	28	2	3	4	1	2	1	3	3	7	11	10	1	6	8	14
PT4140	09V	277	162	28	2	3	4	1	2	1	3	3	7	11	10	1	6	8	14
PT4737	10A	644	1282	22	5	2	9	5	1	2	5	2	7	7	4	2	10	1	27
WL1395	10A	649	97	22	5	2	10	5	1	2	6	6	5	7	4	2	10	1	27
PT450	10A	648	97	22	5	2	10	5	1	2	5	6	5	7	4	2	10	1	27
PT1345	10A	650	97	22	5	2	11	5	1	2	5	6	5	7	4	2	10	1	27
PT3485	11A	11	408	13	1	2	1	1	1	1	2	1	2	5	6	12	16	3	14
WL1344	11A	11	408	13	1	2	1	1	1	1	2	1	2	5	6	12	16	3	14
PT510	11A	30	62	57	1	2	1	6	1	1	2	1	2	5	29	12	16	3	14
PT476	11A	30	62	57	1	2	1	6	1	1	2	1	2	5	29	12	16	3	14
WL555	11A	205	889	12	2	2	8	6	1	2	1	6	8	10	2	16	1	26	107

Key	Serotype	MT	ST	PFGE	MLVA profiles, BOX locus								MLST profiles						
					1	2	3	4	6	11	12	13	aroe	gdh	gki	recP	spi	xpt	ddl
WL1383	11A	205	889	12	2	2	8	6	1	2	1	6	8	10	2	16	1	26	107
WL586	12B	517	218	14	4	2	1	4	2	2	1	4	10	20	14	1	6	1	29
WL737	12F	517	218	14	4	2	1	4	2	2	1	4	10	20	14	1	6	1	29
PT338	14	790	156	28	7	3	4	1	2	1	3	2	7	11	10	1	6	8	1
PT1570	14	817	156	28	8	3	4	1	2	1	3	2	7	11	10	1	6	8	1
PT3291	14	712	143	28	6	2	4	1	2	1	3	4	7	5	10	18	6	8	1
PT3626	14	771	143	29	7	2	4	1	2	1	3	4	7	5	10	18	6	8	1
PT2737	14	771	143	29	7	2	4	1	2	1	3	4	7	5	10	18	6	8	1
DCC2508	14	790	156	33	7	3	4	1	2	1	3	2	7	11	10	1	6	8	1
PT4034	14	374	17	11	3	2	3	2	2	1	10	5	1	5	4	11	9	3	47
PT2667	14	374	17	11	3	2	3	2	2	1	10	5	1	5	4	11	9	3	47
PT3451	14	425	15	8	3	3	2	3	3	1	9	4	1	5	4	5	5	3	8
PT3438	14	425	15	8	3	3	2	3	3	1	9	4	1	5	4	5	5	3	8
PT952	14	367	9	8	3	2	2	3	2	1	2	5	1	5	4	5	5	1	8
PT356	14	367	9	8	3	2	2	3	2	1	2	5	1	5	4	5	5	1	8
PT5244	14	67	411	8	1	2	7	4	2	1	5	3	2	13	14	4	17	4	14
PT5696	15A	111	2105	40	1	3	4	4	2	1	2	4	2	5	36	12	17	21	4
PT1730	15A	678	63	40	5	3	4	4	2	1	2	4	2	5	36	12	17	21	14
WL1327	15A	35	1956	8	1	2	2	4	2	1	5	3	2	19	2	4	17	4	14
PT2111	15B	208	193	9	2	2	9	3	1	1	1	3	8	10	2	16	1	26	1
PT2901	15B	167	172	3	2	2	2	3	1	2	3	6	7	13	8	6	25	6	8
PT4272	15B	67	411	8	1	2	7	4	2	1	5	3	2	13	14	4	17	4	14
PT5245	15B	67	411	8	1	2	7	4	2	1	5	3	2	13	14	4	17	4	14
WL1313	15C	35	1956	8	1	2	2	4	2	1	5	3	2	19	2	4	17	4	14
WL1254	16F	658	30	54	5	3	1	3	2	1	5	6	1	5	27	20	1	1	1
PT4232	16F	660	30	54	5	3	1	3	3	1	5	6	1	5	27	20	1	1	1
PT2585	16F	660	30	54	5	3	1	3	3	1	5	6	1	5	27	20	1	1	1
PT5686	16F	662	30	61	5	3	1	4	3	1	5	6	1	5	27	20	1	1	1
PT735	17F	147	123	32	1	5	8	5	5	1	5	4	7	2	40	1	10	1	45
WL186	17F	128	123	32	1	3	8	5	4	1	5	4	7	2	40	1	10	1	45
PT2605	18A	737	241	11	6	3	4	2	2	1	1	1	25	31	4	16	32	28	44
PT3401	18A	797	241	21	7	3	4	4	2	1	1	1	25	31	4	16	32	28	44
WL1435	18C	188	1877	12	2	2	5	3	1	1	1	8	10	10	41	16	1	26	1
WL1407	18C	544	1016	23	4	2	7	8	3	1	5	6	5	13	11	4	15	1	19
PT4188	18C	49	113	45	1	2	6	2	2	1	5	1	7	2	1	1	10	1	21

					MLVA profiles, BOX locus								MLST profiles						
Key	Serotype	MT	ST	PFGE	1	2	3	4	6	11	12	13	aroe	gdh	gki	recP	spi	xpt	ddl
PT1348	18C	543	1381	19	4	2	6	4	1	2	6	6	10	11	4	16	15	1	145
WL561	19A	197	847	27	2	2	7	2	2	1	9	6	7	11	4	1	6	112	14
PT4972	19A	281	81	14	2	3	5	2	2	1	1	3	4	4	2	4	4	1	1
PT2574	19A	282	81	14	2	3	5	2	2	1	1	99	4	4	2	4	4	1	1
PT1331	19A	678	63	40	5	3	4	4	2	1	2	4	2	5	36	12	17	21	14
PT2542	19A	678	63	40	5	3	4	4	2	1	2	4	2	5	36	12	17	21	14
PT2679	19A	785	230	47	7	3	2	7	1	1	6	3	12	19	2	17	6	22	14
WL677	19A	768	276	68	7	2	2	6	99	1	6	3	2	19	2	17	6	22	14
PT5360	19A	768	276	68	7	2	2	6	99	1	6	3	2	19	2	17	6	222	14
PT2436	19A	768	276	68	7	2	2	6	99	1	6	3	2	19	2	17	6	22	14
PT5687	19A	767	276	9	7	2	2	6	1	1	6	3	2	19	2	17	6	22	14
WL776	19A	761	1026	102	6	99	9	3	2	1	1	1	10	16	32	1	15	28	31
PT5041	19A	190	193	14	2	2	5	6	1	1	1	6	8	10	2	16	1	26	1
PT3188	19A	707	2732	27	5	4	9	99	1	1	1	8	7	60	9	8	10	3	29
PT4288	19A	700	1151	40	5	4	5	99	2	1	1	8	7	60	9	8	6	3	29
PT3171	19A	756	1151	40	6	4	5	99	2	1	1	9	7	60	9	8	6	3	29
PT2808	19A	289	888	20	2	3	8	7	1	1	1	1	8	74	19	15	6	40	26
PT5094	19A	74	416	43	1	2	7	5	2	2	5	5	1	13	14	4	17	51	14
PT4253	19A	83	416	9	1	2	8	5	2	2	5	5	1	13	14	4	17	51	14
PT2757	19A	69	416	9	1	2	7	4	2	2	5	5	1	13	14	4	17	51	14
PT2752	19A	69	416	9	1	2	7	4	2	2	5	5	1	13	14	4	17	51	14
PT14	19A	69	416	9	1	2	7	4	2	2	5	5	1	13	14	4	17	51	14
PT518	19A	178	994	24	2	2	3	5	2	1	10	1	5	5	62	5	6	11	14
WL290	19A	178	994	24	2	2	3	5	2	1	10	1	5	5	62	5	6	11	14
PT1527	19A	743	1801	24	6	3	5	3	2	1	9	2	2	5	4	10	1	1	1
PT3113	19A	743	1801	19	6	3	5	3	2	1	9	2	2	5	4	10	1	1	1
PT516	19F	678	1149	40	5	3	4	4	2	1	2	4	2	5	36	12	1	21	14
PT464	19F	678	1149	40	5	3	4	4	2	1	2	4	2	5	36	12	1	21	14
PT4815	19F	785	230	48	7	3	2	7	1	1	6	3	12	19	2	17	6	22	14
PT584	19F	228	1228	12	2	2	9	5	1	1	1	6	7	10	2	16	1	26	1
WL101	19F	421	391	14	3	3	2	2	2	2	7	1	7	14	4	12	1	20	14
PT332	19F	440	177	18	3	3	6	99	2	2	7	1	7	14	4	12	1	1	14
PT4099	19F	456	179	30	3	3	11	99	2	2	7	1	7	14	40	12	1	1	14
PT4574	19F	295	179	30	2	3	10	99	2	2	7	1	7	14	40	12	1	1	14
PT1282	19F	297	179	32	2	3	11	99	2	2	7	1	7	14	40	12	1	1	14

					MLVA profiles, BOX locus								MLST profiles						
Key	Serotype	MT	ST	PFGE	1	2	3	4	6	11	12	13	aroe	gdh	gki	recP	spi	xpt	ddl
DCC98	19F	275	177	36	2	3	3	99	2	1	7	1	7	14	4	12	1	1	14
PT1809	19F	852	1283	66	99	3	4	2	2	1	5	10	15	16	96	5	6	1	26
WL845	19F	854	236	67	99	3	8	3	1	1	2	1	15	16	19	15	6	20	26
DCC2659	19F	640	87	58	5	2	6	5	3	1	11	2	5	5	7	7	8	5	4
PT4313	19F	636	89	58	5	2	5	5	1	1	10	6	5	5	7	7	8	5	1
PT3815	19F	636	89	58	5	2	5	5	1	1	10	6	5	5	7	7	8	5	1
PT712	19F	359	1487	16	3	2	1	3	2	1	9	2	1	5	4	12	49	3	8
WL1375	19F	556	309	13	4	2	19	3	2	1	10	4	8	10	2	5	9	48	6
PT5137	20	784	230	70	7	3	2	6	99	1	6	3	12	19	2	17	6	22	14
PT505	21	228	1877	12	2	2	9	5	1	1	1	6	10	10	41	16	1	26	1
PT5471	21	230	1877	12	2	2	9	8	1	1	1	7	10	10	41	16	1	26	1
PT4071	22F	302	433	41	2	4	1	1	2	1	1	4	1	1	4	1	18	58	17
WL183	23A	835	42	28	99	2	5	5	2	1	9	3	1	8	9	9	6	4	6
PT5298	23A	836	42	28	99	2	5	5	2	1	9	6	1	8	9	9	6	4	6
PT829	23B	800	439	62	7	3	5	2	2	2	4	2	1	8	9	2	6	4	6
PT380	23B	819	439	9	8	3	5	2	2	2	5	2	1	8	9	2	6	4	6
PT4450	23F	279	81	14	2	3	4	2	2	1	1	99	4	4	2	4	4	1	1
PT1283	23F	279	81	14	2	3	4	2	2	1	1	99	4	4	2	4	4	1	1
PT1309	23F	765	338	26	7	2	2	2	3	2	6	4	7	13	8	6	1	6	8
DCC1587	23F	622	338	3	5	2	2	2	3	2	5	4	7	13	8	6	1	6	8
PT2844	23F	641	338	4	5	2	7	2	3	2	1	6	7	13	8	6	1	6	8
PT5527	23F	710	338	94	6	2	2	2	99	2	5	4	7	13	8	6	1	6	8
PT390	23F	234	242	18	2	2	11	4	4	2	5	4	15	29	4	21	30	1	14
PT239	23F	141	3394	46	1	4	5	10	0	2	4	9	1	13	9	2	6	4	6
PT1592	23F	7	33	49	1	1	5	4	0	2	4	2	1	8	1	2	6	4	6
PT766	23F	7	33	49	1	1	5	4	0	2	4	2	1	8	1	2	6	4	6
PT803	23F	7	33	49	1	1	5	4	0	2	4	2	1	8	1	2	6	4	6
PT2942	24F	780	230	47	7	3	2	2	1	1	6	3	12	19	2	17	6	22	14
PT1721	24F	568	72	25	4	3	7	5	0	1	5	4	2	13	2	4	9	4	1
PT460	29	723	1342	31	6	2	8	1	1	1	3	1	2	12	94	1	6	28	14
PT5412	29	769	198	53	7	2	3	3	0	1	1	1	8	13	4	8	6	22	34
PT5572	31	87	1766	10	1	2	9	3	1	1	2	2	1	5	29	1	46	14	18
PT1287	31	10	533	11	1	1	10	5	1	1	2	1	1	5	29	1	46	1	18
PT1274	31	95	1766	29	1	2	11	3	1	1	2	2	1	5	29	1	46	14	18
PT1581	31	94	2002	9	1	2	11	2	1	1	2	2	1	5	135	1	46	14	18

					MLVA profiles, BOX locus								MLST profiles						
Key	Serotype	MT	ST	PFGE	1	2	3	4	6	11	12	13	aroe	gdh	gki	recP	spi	xpt	ddl
WL386	33F	164	1367	13	2	2	2	1	1	2	1	2	2	16	29	18	42	3	18
DCC2253	33F	173	717	37	2	2	2	6	0	2	2	1	5	35	29	1	45	39	18
PT2655	33F	173	717	37	2	2	2	6	0	2	2	1	5	35	29	1	45	39	18
PT2673	33F	173	717	37	2	2	2	6	0	2	2	1	5	35	29	1	45	39	18
PT5660	33F	173	717	37	2	2	2	6	0	2	2	1	5	35	29	1	45	39	18
PT3341	34	739	1046	27	6	3	4	3	0	2	5	4	15	5	2	1	9	1	18
WL1402	34	92	478	32	1	2	10	1	1	1	1	3	45	13	6	12	9	14	1
PT2552	34	534	2001	30	4	2	6	1	1	1	1	1	5	125	6	1	9	14	14
PT1573	34	534	2001	30	4	2	6	1	1	1	1	1	5	125	6	1	9	14	14
PT5504	34	538	2001	65	4	2	6	1	2	1	1	1	5	125	6	1	9	14	14
PT2398	35B	304	1955	41	2	4	1	3	2	1	1	4	1	118	4	1	18	58	17
PT3419	35F	97	446	41	1	2	15	5	2	1	2	2	5	7	4	19	10	40	27
PT1314	35F	97	446	41	1	2	15	5	2	1	2	2	5	7	4	19	10	40	27
PT5282	35F	316	1368	22	2	4	5	3	1	2	9	3	7	5	4	5	42	92	79
PT3491	35F	316	1368	22	2	4	5	3	1	2	9	3	7	5	4	5	42	92	79
PT3501	37	4	447	2	1	1	2	1	2	2	2	2	29	33	19	1	36	22	31
WL397	38	127	393	27	1	3	7	6	99	2	2	4	10	43	41	18	13	49	6
WL235	38	126	393	27	1	3	7	6	3	2	2	4	10	43	41	18	13	49	6
PT4264	39	156	1126	17	2	1	5	4	2	2	1	8	7	5	92	16	6	1	31
WL1215	42	304	1955	41	2	4	1	3	2	1	1	4	1	118	4	1	18	58	17
PT1683	NT	790	156	28	7	3	4	1	2	1	3	2	7	11	10	1	6	8	1
PT5002	NT	520	1156	1	4	2	2	2	2	1	2	1	2	13	2	29	91	19	59
PT4222	NT	520	1156	1	4	2	2	2	2	1	2	1	2	13	2	29	91	19	59
PT4014	NT	520	1153	52	4	2	2	2	2	1	2	1	2	13	2	29	91	19	141
DCC2648	NT	500	941	109	3	99	2	3	99	1	2	6	8	10	15	27	2	28	71
DCC2787	NT	501	941	109	3	99	2	3	99	1	2	7	8	10	15	27	2	28	71
WL992	NT	812	344	111	7	99	2	9	99	1	2	2	8	37	9	29	2	12	53
LgSt142	NT	812	344	112	7	99	2	9	99	1	2	2	8	37	9	29	2	12	53
DCC2119	NT	812	344	113	7	99	2	9	99	1	2	2	8	37	9	29	2	12	53
DCC689	NT	810	344	113	7	6	2	9	99	1	2	2	8	37	9	29	2	12	53
PT780	NT	812	344	113	7	99	2	9	99	1	2	2	8	37	9	29	2	12	53
DCC2489	NT	812	344	113	7	99	2	9	99	1	2	2	8	37	9	29	2	12	53
DCC2430	NT	810	344	113	7	6	2	9	99	1	2	2	8	37	9	29	2	12	53
PT5466	NT	823	344	113	9	99	2	2	99	1	4	2	8	37	9	29	2	12	53
DCC2367	NT	335	344	113	2	99	2	9	99	1	2	2	8	37	9	29	2	12	53

					MLVA profiles, BOX locus								MLST profiles						
Key	Serotype	MT	ST	PFGE	1	2	3	4	6	11	12	13	aroe	gdh	gki	recP	spi	xpt	ddl
PT3044	NT	812	344	113	7	99	2	9	99	1	2	2	8	37	9	29	2	12	53
PT806	NT	812	344	113	7	99	2	9	99	1	2	2	8	37	9	29	2	12	53
PT1608	NT	812	344	114	7	99	2	9	99	1	2	2	8	37	9	29	2	12	53
WL1210	NT	812	344	114	7	99	2	9	99	1	2	2	8	37	9	29	2	12	53
PT191	NT	812	344	115	7	99	2	9	99	1	2	2	8	37	9	29	2	12	53
PT4671	NT	810	344	116	7	6	2	9	99	1	2	2	8	37	9	29	2	12	53
DCC2362	NT	335	344	116	2	99	2	9	99	1	2	2	8	37	9	29	2	12	53
PT944	NT	505	344	117	3	99	2	9	99	1	2	2	8	37	9	29	2	12	53
PT998	NT	505	344	117	3	99	2	9	99	1	2	2	8	37	9	29	2	12	53
DCC2870	NT	786	897	118	7	3	2	9	99	1	2	2	8	37	36	29	2	12	14
DCC2879	NT	786	897	118	7	3	2	9	99	1	2	2	8	37	36	29	2	12	14
WL1530	NT	812	344	120	7	99	2	9	99	1	2	2	8	37	9	29	2	12	53
WL1514	NT	812	344	120	7	99	2	9	99	1	2	2	8	37	9	29	2	12	53
PT5474	NT	812	344	120	7	99	2	9	99	1	2	2	8	37	9	29	2	12	53
PT2987	NT	812	344	47	7	99	2	9	99	1	2	2	8	37	9	29	2	12	53
WL1084	NT	498	448	110	3	99	2	3	3	1	2	6	8	5	2	27	2	11	71
PT4812	NT	520	1278	39	4	2	2	2	2	1	2	1	2	6	4	29	91	19	147
PT673	NT	504	1618	74	3	99	2	4	7	1	1	4	2	98	9	65	107	47	14

MT, MLVA type. ST, Sequence Type.
